# When impact trials are not feasible: alternatives to study the impact of prediction models on clinical practice

**DOI:** 10.1093/ndt/gfae170

**Published:** 2024-07-17

**Authors:** Roemer J Janse, Vianda S Stel, Kitty J Jager, Giovanni Tripepi, Carmine Zoccali, Friedo W Dekker, Merel van Diepen

**Affiliations:** Department of Clinical Epidemiology, Leiden University Medical Center, Leiden, The Netherlands; ERA Registry, Department of Medical Informatics, Amsterdam UMC location University of Amsterdam, Amsterdam, The Netherlands; Amsterdam Public Health Research Institute, Quality of Care, Amsterdam, The Netherlands; ERA Registry, Department of Medical Informatics, Amsterdam UMC location University of Amsterdam, Amsterdam, The Netherlands; Amsterdam Public Health Research Institute, Quality of Care, Amsterdam, The Netherlands; CNR-IFC, Clinical Epidemiology of Renal Diseases and Hypertension, Reggio Calabria, Italy; CNR-IFC, Clinical Epidemiology of Renal Diseases and Hypertension, Reggio Calabria, Italy; Department of Clinical Epidemiology, Leiden University Medical Center, Leiden, The Netherlands; Department of Clinical Epidemiology, Leiden University Medical Center, Leiden, The Netherlands

**Keywords:** impact, impact trial, kidney disease, nephrology, prediction model

## Abstract

Patients with kidney disease have an uncertain future, with prognosis varying greatly per patient. To get a better idea of what the future holds and tailor interventions to the individual patient, prediction models can be of great value. Before a prediction model can be applied in practice, its performance should be measured in target populations of interest (i.e. external validation) and whether or not it helps improve clinical practice (i.e. whether it impacts clinical practice) should be determined. The impact would ideally be determined using an impact trial, but such a trial is often not feasible, and the impact of prediction models is therefore rarely assessed. As a result, prediction models that may not be so impactful may end up in clinical practice and impactful models may not be implemented due to a lack of impact studies. Ultimately, many prediction models end up never being implemented, resulting in much research waste. To allow researchers to get an indication of a prediction model's impact on clinical practice, alternative methods to assess a prediction model's impact are important. In this paper, we discuss several alternatives, including interviews, case-based surveys, decision comparisons, outcome modelling, before–after analyses and decision curve analyses. We discuss the general idea behind these approaches, including what information can be gathered from such studies and important pitfalls. Lastly, we provide examples of the different alternatives.

## BACKGROUND

### Prediction modelling

Patients with kidney disease have an uncertain future with prognosis varying greatly per patient. To get a better idea of what the future holds and tailor interventions to the individual patient, prediction models can be of great value. A prediction model aims to indicate the prognosis of an individual patient, most often as a risk estimate (e.g. a 19% chance of advancing to kidney failure within 2 years) [1]. These models can be used with a decisive or assistive aim (Box 1) [2].

Box 1. **Decisive versus assistive models**

A decisive model has a cut-off (i.e. a decision threshold) above which a certain intervention should be given. In other words, such a model decides who gets an intervention based on the prediction. This is often protocolized, with the threshold based on clinical expertise or an impact study.

 In contrast, an assistive model can also be used for decision-making, but does not dictate it. Instead, an assistive model is meant to inform the healthcare provider and the patient. This may also help in deciding a treatment strategy, but leaves more room to also use other information. The healthcare provider and the patient may choose to incorporate the prediction into their decision-making, but can also leave it aside, or give priority to other information.

 For instance, using the Kidney Failure Risk Equation, a healthcare provider could decide to refer all patients with a risk >2% to the nephrologist (decisive approach) or to take into account their increased risk together with other risk factors (e.g. age, lifestyle) in deciding whether referral is needed (assistive approach).

Prediction models are developed using multiple variables that are weighted to determine the final prediction (e.g. the risk estimate) [3]. The prediction model will perform best in the population where it was developed, as it was fine-tuned to that specific population [4]. However, the model is most often meant to be used for new patients, meaning that its performance should also be evaluated in these new populations. This is termed external validation, and is an essential aspect of developing a prediction model [5]. Although external validation used to be rare for prediction models, in recent years there has been an increase in external validation studies being performed [5, 6].

To be of value for healthcare, a prediction model should have a beneficial impact on clinical practice, which may be broadly interpreted and can be expressed in different ways, such as improvement in clinical outcomes, patient satisfaction, certainty of decisions and improved decision-making, cost-effectiveness, healthcare resource utilization and more. Nonetheless, good performance of a prediction model does not automatically mean it will beneficially impact clinical practice [2]. The prediction model might be implemented but not used at all for multiple reasons [7]. For instance, low trust in the prediction model or a lack of (perceived) added value of the model would lead to little use of the prediction model, meaning it cannot impact clinical practice. Additionally, no effective treatment might be available for identified high-risk patients. It is also highly important to prevent implementation of harmful prediction models, which may be the case if model performance is not adequate in the population where it is used. Thus, the study of a prediction model's impact on clinical practice is of paramount importance [8].

In the ideal situation, the impact of a prediction model would be studied with a randomized controlled trial (known as the impact trial). This design allows us to determine the causal effect of implementation of a prediction model on our outcomes of interest. Because a learning effect might occur in the healthcare provider (i.e. what the healthcare provider learned for one patient, they will also apply to another patient), patients cannot be randomized on the individual level [2, 3]. Additionally, healthcare providers within the same centre may share their experiences of using the model with each other [2, 3]. Therefore, a cluster randomized controlled trial at the centre level is best suited [2, 3]. For instance, one impact trial has been set up to study the impact of implementing the Kidney Failure Risk Equation (KFRE) on multiple outcomes, such as treatment with renin–angiotensin system inhibitors (RASi) and decline in estimated glomerular filtration rate (eGFR), using a cluster randomized controlled design [9]. However, impact trials require large sample sizes, are expensive and might not be funded without prior indication of a prediction model's impact. Moreover, for a decisive prediction model, a decision threshold is needed. Nonetheless, the optimal decision threshold might not be known. Ideally, the impact trial would then consist of multiple intervention arms with differing decision thresholds to study the impact of the prediction model over a range of different decision thresholds.

Currently, impact studies are rarely performed [6]. As many papers have stressed the importance of measuring impact [1–3, 7], in this paper, we will discuss alternatives to the (often infeasible) impact trial to study the impact of a prediction model. Per alternative, we describe the underlying idea, its considerations and what dimensions of impact it can study, and give examples from the medical literature that used these alternatives.

## ALTERNATIVES TO THE IMPACT TRIAL

As the field of impact studies for prediction models is relatively new and still evolving, there is no established set of alternatives to study impact beyond the impact trial. However, we may utilize and adapt existing techniques or develop new alternative methods to assess the impact of a prediction model on clinical practice (Table 1). Different approaches provide varying indications of the impact, ranging from what would hypothetically happen to what actually happened (albeit not directly attributable to the prediction model) (Table 2). The fact that these approaches might not be as conclusive as the impact trial notwithstanding, they may still provide valuable results when an impact trial is not feasible or in addition to an impact trial (e.g. perceived barriers). The list of alternatives is however not exhaustive. Other alternatives, such as Markov modelling [10] and cross-sectional randomization [2], have been discussed elsewhere [3, 7].

**Table 1:** Overview of different alternatives to study the impact of a prediction model with a short description and whether they can be used for assistive or decisive models.


**Alternative**	**Description**	**Assistive/decisive model**	**Considerations**	**Examples**

Impact trial	Cluster randomized controlled trial randomizing hospitals to use or not use the prediction model	Both	Expensive, not always feasible	[9]
Interviews	Interview patients and healthcare providers to know their perspectives	Both	Intentions do not automatically translate into actions	Preprint by Bergeron et al. (2024)
Case-based survey	Survey with cases to determine how healthcare providers would change their decision	Assistive	Healthcare providers might act differently in a research setting than in a real-world setting	[12‐14]
Comparison of decisions	Use existing data to study how often an intervention took place. Compare to how often intervention would have taken place if prediction model was used	Decisive	Some decisions might not change even under different decision rules, e.g. due to contra-indications	[15, 16, 27]
Modelling of outcomes	Use existing data to study outcomes between individuals receiving and not receiving the intervention according to the prediction model. Then compare these individuals with their counterparts that received or did not receive the intervention contrary to the prediction model	Decisive	Large sample size required, risk of non-positivity	
Before–after analysis	Study outcomes before and after implementation of a prediction model	Both	Changes might be attributable to time	[15, 23]
Decision curve analysis	Compare the net benefit of a prediction model against different strategies over a range of preferences regarding the harm–benefit ratio	Decisive	Needs to be compared with clear decision rules, assumed that everyone above the threshold receives treatment and it (fully) alleviates the disease	[26, 27]

**Table 2:** Overview of the different dimensions of impact one can investigate using each alternative method; this list is not exhaustive.

**Alternative**	**Changes in HPs decisions**	**Changes in HPs confidence in a decision**	**Clinical outcomes**	**Cost-effectiveness**	**Expected impact**	**Healthcare utilization**	**HPs willingness to use the model**	**Impact on patients’ lives**	**Implementation barriers**	**Patient satisfaction**
Impact trial	✔	✔	✔	✔	✔	✔	✔	✔	✔	✔
Interviews					✔		✔	✔	✔	✔
Case-based survey	✔	✔			✔		✔		✔	
Comparison of decisions	✔									
Modelling of outcomes	✔		✔	✔		✔				
Before–after analysis	✔		✔	✔		✔				
Decision curve analysis	✔		✔							

HP, healthcare provider.

### Interviews

To identify how patients and healthcare providers value the implementation of a prediction model in clinical practice, a qualitative approach could be used [11]. For instance, semi-structured interviews could generate a comprehensive perspective of the impact of a prediction model. This could be a hypothetical perspective, inquiring about how healthcare providers expect a prediction model to impact their practice while it is not actually implemented (yet), or based on an already implemented model. In the latter case, patients could also be asked about their experiences with the provided information and how this affects different aspects of their lives (e.g. dealing with uncertainty in the future). Additionally, interviews explore the perceived barriers to using the prediction model in clinical practice. An alternative to semi-structured interviews would be focus groups, which allow interaction between individuals (possibly a mixture of healthcare providers and patients) to build on individual ideas and get a more refined idea of a prediction model's impact.

This approach is advantageous because it can identify many perspectives and give a comprehensive overview of how a prediction model could/does impact clinical practice. It may do so for models with both a decisive and an assistive aim. However, it cannot provide an answer regarding improvement in clinical outcomes, nor do intentions (e.g. a healthcare provider wanting to use a prediction model) automatically translate into actions (healthcare provider actually using the prediction model).

This alternative was used by Bergeron *et al.* in a preprint titled ‘Nephrology Providers’ Perspective and Use of Mortality Prognostic Tools in Dialysis Patients’ (2024). The authors applied three mortality prediction models for patients with kidney failure and showed the results to 10 healthcare providers. Then, using semi-structured interviews, they identified barriers for implementation. These included concerns for generalizability, healthcare providers’ belief in their own judgement, lack of clarity on how these tools were developed and a lack of available time. The authors can leverage this information to improve model uptake and trust, thereby allowing the model to impact clinical practice.

### Case-based survey

Another method to determine the impact of a prediction model is performing a case-based survey. This method, which is best suited for assistive models, uses clinical cases to understand how healthcare providers would be impacted in their decision-making based on a prediction model. Cases can consist of situations in which there is no clear decision, and comparisons can be made between what healthcare providers would decide under standard care and what they would decide if they additionally received information from the prediction model. Moreover, such a survey can also inquire about how the prediction model impacts a healthcare provider's confidence regarding the treatment decision with and without the knowledge of an individual's predicted risk.

The survey is advantageous in the sense that a large group of healthcare providers can be reached relatively easy. Additionally, it allows for the quantification of the impact of the prediction model. Nonetheless, it should be noted that these results do not say anything about what would actually happen: the cases are presented in a research setting, and in a real-world situation healthcare providers may still act differently. Moreover, the impact on outcomes of interest, such as clinical endpoints and patient satisfaction, cannot be studied.

This design was used by Schutter *et al.* to determine the impact of the PRE-IMAGE prediction model, which predicts the risk of an adverse outcome prior to kidney transplantation [12]. Schutter *et al.* surveyed 60 Dutch nephrologists using six hypothetical cases inspired by real-world cases. The nephrologists decided twice on each case: once without knowing the predicted risk and once whilst knowing the predicted risk. The authors investigated interobserver variability (the variability in whether the nephrologists accepted a kidney), the kidney acceptance rate and the certainty nephrologists had in their decision. With information from the prediction model, the interobserver variability decreased, the acceptance rate of kidneys changed and nephrologists felt more certain about their decision. Although from this study, we cannot know whether these nephrologists would have acted differently in a real-life setting and whether their changed decisions would have improved outcomes, we can say that the prediction model did impact their decision-making for these hypothetical cases, demonstrating potential impact.

Surveys may also be used to determine the perspectives of healthcare providers and patients on prediction models and their willingness to receive and use information from prediction models. Such information is important for implementing a model so that it may have a positive impact, as positive attitudes of the users towards prediction models are a prerequisite to their use in clinical practice. To this end, Kotsis *et al.* surveyed 54 German nephrologists to determine current use, helpfulness and willingness to use prediction models [13]. Similarly, van der Horst *et al.* surveyed 126 patients with chronic kidney disease and 50 nephrologists, finding that prediction models were infrequently used to discuss patients’ future and that patients differed in their preference of knowing about their individualized predicted risk for different outcomes [14].

### Comparison of decisions

Although case-based surveys can be used for assistive models, a better alternative is available for decisive models. Given that decisive models aim to direct interventions, a comparison can be made between whether healthcare providers intervene and whether the prediction model would have prompted an intervention. We can determine how often an intervention took place using available data from clinical practice. Subsequently, we can calculate predictions for individuals and determine whether they would have received the intervention based on that prediction. Then, we can compare whether the intervention would have been received more or less often if the prediction model was used, as compared with what decisions were actually made by the healthcare providers. For instance, with a hypothetical prediction model that directs receiving RASi treatment after acute kidney injury if the predicted risk of mortality is 30% or higher, we can compare how often RASi was actually prescribed and how often the prediction model would have indicated a RASi prescription.

Notably, this does not say whether (or not) intervening in certain individuals based on the prediction model leads to improved health outcomes. However, it provides an indication of whether the prediction model would change anything in clinical practice. Additionally, if the data are present, such as in the form of electronic health records, the comparison is relatively easy to make (dependent on the variables used in the prediction model).

In a Belgian study, Philipse *et al.* retrospectively calculated the Kidney Donor Risk Index (KDRI) for all deceased donor kidneys in their hospital between 2010 and 2013 [15]. The authors noted that the KDRI in their hospital was generally low (indicating a relatively low risk of graft failure), including for kidneys they discarded. The authors then concluded that they might be declining too many offers. This also indicates that the KDRI would lead to different decisions regarding transplanting kidneys as compared with their decision-making system prior to the KDRI. Thus, the authors could conclude that the KDRI would indeed change decision-making in their clinical practice if it were to be implemented (and used for decision-making), although they could not study whether that change would lead to improved outcomes.

As a second example, Bhachu *et al.* compared the UK National Institute of Health and Care Excellence (NICE) 2014 CKD guidelines for nephrologist referral with referral based on a 5-year KFRE risk >3% [16]. Among 39 476 patients with CKD stage 3–5, they found that using the KFRE referral rule would mean that 2386 individuals would not be referred contrary to the NICE guidelines and that 3483 individuals would be referred contrary to the NICE guidelines.

### Modelling of outcomes

We may also study whether this changed decision-making would lead to improved outcomes. For such a study, we use data in which the prediction model was not used but will pretend it was used. Using the RASi after AKI example, we can create four groups:

(i) individuals who did not receive RASi and had a predicted risk <30% (concordant non-assignment);
(ii) individuals who received RASi and had a predicted risk <30% (discordant assignment);
(iii) individuals who did not receive RASi and had a predicted risk ≥30% (discordant non-assignment);
(iv) individuals who received RASi and had a predicted risk ≥30% (concordant assignment).

In our ideal impact trial, we would investigate the difference in outcomes between the individuals who are assigned treatment and not assigned treatment according to the prediction model. In our observational data, this would mean studying the effect of receiving or not receiving RASi according to the prediction model [groups (i) and (iv)] on mortality. However, because the groups are stratified on their risk of mortality, they likely differ on other important risk factors for mortality, which may cause confounding [17]. Confounding and other biases in such an observational study [18] may be alleviated by trying to emulate our ideal impact trial using observational data [19]. It should be noted that an intervention based solely on a prediction model's decision complicates confounding adjustment. Because certain confounders might also be predictors in the prediction model, they may occur only in the treated (or only in the untreated) individuals. This leads to mathematical complexities that impede making causal statements. This is called non-positivity. Although strategies exist to alleviate non-positivity [20], it is important to be aware of the risk. If the analysis is performed correctly and treatment is known to be effective (such as RASi), we would expect no difference in the outcome, as the individuals receiving treatment are then correctly treated to reduce their risk of the outcome. If treatment does not completely alleviate the risk of the outcome, we should also compare all discordant non-assigned individuals with concordant assigned individuals [groups (iii) and (iv)] to determine whether treatment at high risk has a protective effect.

However, the individuals for whom treatment would change if the prediction would be implemented are those who were not treated according to the prediction model [discordant individuals: groups (ii) and (iii)]. We want to know whether for them outcomes would improve too upon implementation of the prediction model. To do this, we make two additional comparisons: (a) concordant and discordant non-assigned individuals [groups (i) and (ii)] and (b) concordant and discordant assigned individuals [groups (iii) and (iv)]. If these comparisons show large differences between the groups, we might not be able to generalize the results of the concordant comparison to them. Subsequently, we cannot get a good idea of a prediction model's impact, as we would not know how it would impact the individuals for whom treatment would change (i.e. the discordant individuals).

If the costs of different procedures are known and these procedures are also measured in the data (e.g. admissions, outcomes, prescriptions), the cost-effectiveness of the model may also be calculated.

### Before–after analysis

We may also get an idea of the impact of a model by measuring certain outcomes before and after the implementation of a model, or study outcomes that are registered by default (e.g. dialysis complications) for an already implemented model.

This approach allows us to study how relevant outcomes have changed after implementing a prediction model (both assistive and decisive). However, a disadvantage is that we cannot directly attribute these changes to the implementation of the prediction model, as clinical practice may also change over time. To partially alleviate this, a second outcome may be selected that should not be influenced by the prediction model but would be influenced by other changes over time in a similar way to our outcome of interest (i.e. a negative control outcome) [18, 21]. If we do not see a change in this negative control outcome, we can be more certain that any change in the primary outcome of interest is due to the prediction model. An adaptation of the before–after analysis is the on–off analysis, which is discussed elsewhere [2]. Similar to the modelling of outcomes, cost-effectiveness can be determined if costs of procedures, admissions, etc., are also known.

The Belgian study from Philipse *et al.* also studied the impact of the KDRI by comparing the transplant rate and the reasons for discarding kidneys before and after KDRI implementation [15]. The authors found that transplantation increased and that predictors used for calculating the KDRI were less often a reason to decline a kidney for transplantation.

The KDRI can be translated into the Kidney Donor Profile Index (KDPI): a measure of donor kidney quality relative to other available kidneys with higher values indicating a higher risk of graft failure [22]. Bae *et al.* studied the changes in kidney discard rate before and after introducing the KDPI [23]. In a population of kidney transplant recipients from the USA, the authors studied the number of kidneys discarded and the survival benefit after introducing the KDPI. Before the KDPI, kidneys could be classified as standard criteria donors (SCD) (generally kidneys with a lower risk of graft failure) or extended criteria donors (generally higher risk of graft failure). The authors described that SCD kidneys with a high KDPI (i.e. kidneys with a lower risk of graft failure in the old system but a higher risk of graft failure according to the KDPI) were more often discarded. Nonetheless, individuals transplanted with SCD kidneys with a high KDPI still showed decreased long-term mortality compared with individuals remaining on the waiting list. The authors conclude from this that these kidneys (SCD with high KDPI) would have been better accepted for transplantation as they still provide a survival benefit over no transplantation, despite the high risk of graft failure.

### Decision curve analysis

The last alternative method to study impact we discuss, the decision curve, offers an accessible method of determining the potential impact of a decisive prediction model. In a decision curve analysis, we calculate the net benefit of using a prediction model to direct an intervention. Imagine we use the KFRE to estimate an individual's 2-year risk of kidney failure [24]. Using this estimated risk, we decide that anyone with a 2-year risk of 20% or above (the decision threshold) should get vascular access, as they are likely to start dialysis within a year.

We can then calculate the net benefit for the prediction model. The net benefit comprises true positives (received vascular access and started dialysis within a year), false positives (received vascular access but did not start dialysis within a year) and a harm–benefit ratio. The harm–benefit ratio portrays the personal preference of a patient or a healthcare provider for whether an unnecessary intervention or wrongly not intervening is more important: for instance, a healthcare provider might rather have five patients get vascular access for dialysis too early to make sure one patient does not get vascular access too late. This can be portrayed as a harm–benefit ratio of 16.7% or as odds (1:5). Given that per individual, the preferred harm–benefit ratio might differ, there is relevance in showing the net benefit across a range of threshold harm–benefit ratios. The decision curve also contains two standard strategies (treat everyone and treat no one) to which the prediction model can be compared. Thus, the decision curve analysis allows us to determine the harm–benefit ratios the prediction model would give the highest net benefit (especially over the default strategies). A more elaborate explanation of decision curve analyses is available elsewhere [25].

Although decision curve analysis is suitable where clear guidelines or prior prediction models are available, there are also situations where it cannot compare the prediction model against actual clinical practice. Moreover, when using decision curve analysis to measure impact, we assume that being above the decision threshold means receiving the treatment and that the intervention (fully) alleviates the disease. However, this is not always the case as treatment might not be 100% effective or work for each individual.

**Figure 1:**
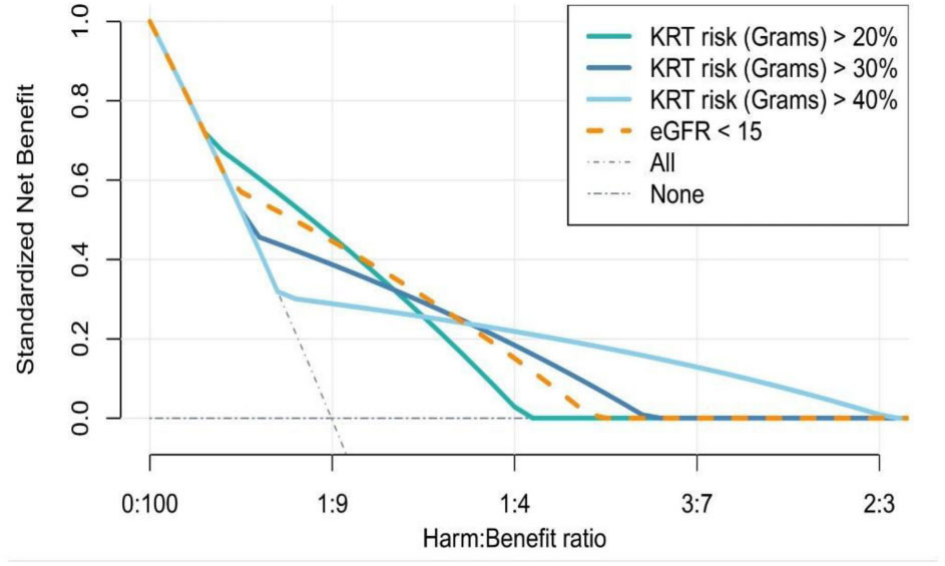
Decision curve analysis for different decision thresholds with the Grams prediction model, an eGFR <15 mL/min/1.73 m^2^, and the default strategies of treat all and treat none. Source: adapted from Ramspek CL *et al*., ‘Predicting kidney failure, cardiovascular disease and death in advanced CKD patients’ *Kidney Int Rep* 2022 Aug 2;7(10):2230–2241 [26]. Licensed under CC-BY 4.0. Original copyright (2022) by the International Society of Nephrology. Published by Elsevier, Inc.

A study performed by Ramspek *et al.* used decision curve analysis to study the impact of the Grams model, which predicts kidney failure and aims to guide vascular access placement [26]. The authors studied the net benefit of using a predicted risk of 20%, 30%, 40% and 50% risk at 2 years as a decision threshold to guide vascular access, also comparing this to the eGFR level of <15 mL/min/1.73 m^2^ as a decision threshold. They defined true positives as individuals above the decision threshold that did indeed start kidney replacement therapy (KRT) within a year and false positives as individuals above the decision threshold that did not start KRT within a year. The authors found that the Grams prediction model gave a higher net benefit at almost all harm–benefit ratios than the default strategies or the eGFR threshold of <15 mL/min/1.73 m^2^ (Fig. 1). Lundström *et al.* also used this approach to study the impact of the KFRE for guiding vascular access placement [27]. In addition to performing a decision curve analysis, the authors also compared how treatment would change using the KFRE compared with eGFR levels (comparison of decisions). By combining the alternatives of decision curve analysis and comparison of decisions, they strengthened their case that the KFRE would positively impact clinical practice.

## CONCLUSION

Although an impact trial allows us to conclusively determine the impact of a prediction model on clinical practice, such a trial is not always feasible. We discussed several alternatives, albeit that they all come with their own limitations. These alternatives allow us to study impact in different ways and together with the impact trial give us an extensive toolbox to study a prediction model's impact. No combination of these alternatives is the ideal combination for any given situation. When we want to make a compelling case to implement a prediction model in clinical practice, we must first determine where we want to make a difference (e.g. clinical outcomes). Then, we can choose alternatives capable of studying this, specific to our own situation. Other alternatives can then solidify our case. For instance, interviews with healthcare providers will likely yield areas where uptake can be improved (e.g. willingness). As a last note, the list of these alternatives is not exhaustive; in the future we may come up with more ways to study the impact of a prediction model.

## Data Availability

No data were used in this study.
